# Defect-mediated selective hydrogenation of nitroarenes on nanostructured WS_2_†
†Electronic supplementary information (ESI) available: Additional material characterization and DFT calculation data. See DOI: 10.1039/c9sc03337h


**DOI:** 10.1039/c9sc03337h

**Published:** 2019-09-19

**Authors:** Yifan Sun, Albert J. Darling, Yawei Li, Kazunori Fujisawa, Cameron F. Holder, He Liu, Michael J. Janik, Mauricio Terrones, Raymond E. Schaak

**Affiliations:** a Department of Chemistry and Materials Research Institute , The Pennsylvania State University , University Park , PA 16802 , USA . Email: res20@psu.edu; b Department of Chemical Engineering , The Pennsylvania State University , University Park , PA 16802 , USA . Email: mjj13@psu.edu; c Department of Physics , The Pennsylvania State University , University Park , PA 16802 , USA . Email: mut11@psu.edu; d Department of Materials and Science Engineering , The Pennsylvania State University , University Park , PA 16802 , USA; e Center for 2-Dimensional and Layered Materials , The Pennsylvania State University , University Park , PA 16802 , USA

## Abstract

Transition metal dichalcogenides (TMDs) are well known catalysts as both bulk and nanoscale materials. Two-dimensional (2-D) TMDs, which contain single- and few-layer nanosheets, are increasingly studied as catalytic materials because of their unique thickness-dependent properties and high surface areas. Here, colloidal 2H-WS_2_ nanostructures are used as a model 2-D TMD system to understand how high catalytic activity and selectivity can be achieved for useful organic transformations. Free-standing, colloidal 2H-WS_2_ nanostructures containing few-layer nanosheets are shown to catalyze the selective hydrogenation of a broad scope of substituted nitroarenes to their corresponding aniline derivatives in the presence of other reducible functional groups. Microscopic and computational studies reveal the important roles of sulfur vacancy-rich basal planes and tungsten-terminated edges, which are more abundant in nanostructured 2-D materials than in their bulk counterparts, in enabling the functional group selectivity. At tungsten-terminated edges and on regions of the basal planes having high concentrations of sulfur vacancies, vertical adsorption of the nitroarene is favored, thus facilitating hydrogen transfer exclusively to the nitro group due to geometric effects. At lower sulfur vacancy concentrations on the basal planes, parallel adsorption of the nitroarene is favored, and the nitro group is selectively hydrogenated due to a lower kinetic barrier. These mechanistic insights reveal how the various defect structures and configurations on 2-D TMD nanostructures facilitate functional group selectivity through distinct mechanisms that depend upon the adsorption geometry, which may have important implications for the design of new and enhanced 2-D catalytic materials across a potentially broad scope of reactions.

## Abstract 2

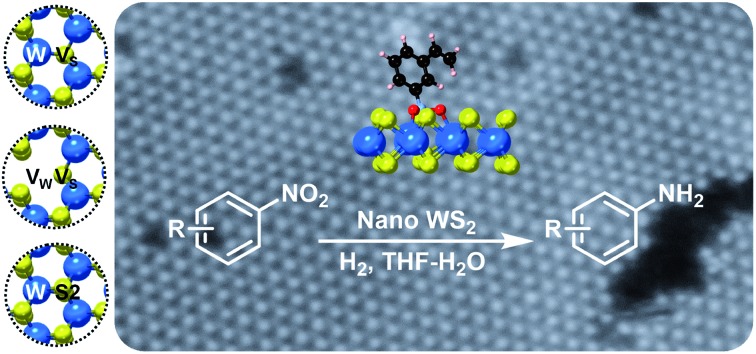
Colloidal 2H-WS_2_ nanostructures catalyze selective hydrogenation of substituted nitroarenes to anilines with molecular hydrogen, where sulfur vacancies and tungsten-terminated edges play key roles in enabling functional group selectivity.

## Introduction

Two-dimensional transition metal dichalcogenide (TMD) materials are active catalysts for a broad range of chemical transformations, including the hydrogen evolution reaction,^1^ the electrochemical reduction of CO_2_,^2^ and various hydrotreating and hydrogenation reactions.^3^–^5^ The near-zero free energy of adsorption of molecular hydrogen on edge and defect sites makes TMDs ideal candidates as active hydrogenation catalysts.^6^ Hydrogenation catalysts that can target one reducible group on a molecule when others are present and accessible are especially important. For example, the selective hydrogenation of substituted nitroarenes is important for accessing aniline compounds that are useful intermediates in pharmaceutical and agrochemical production.^7^

Catalysts that facilitate selective hydrogenation reactions using H_2_ are desirable based on atom economy considerations and the formation of water as the only reduction byproduct.^8^–^10^ Among heterogeneous catalysts, chemically modified noble metals can achieve selective hydrogenation,^11^–^13^ but recent interest in new and low-cost materials has resulted in a growing number of non-platinum group (NPG) transition metal catalysts for such reactions.^14^–^18^ TMD materials are well known NPG hydrogenation catalysts.^19^ The more recent ability to synthesize two-dimensional (2-D) TMDs containing single- and few-layer nanosheets has expanded the catalytic scope of these materials and enabled new capabilities in achieving functional group selectivity. For example, nanostructured MoS_2_ catalyzes the selective hydrogenation of substituted nitroarenes using H_2_ to form their corresponding anilines, but requires transition metal promotors to facilitate the formation of active sites that arise from a significantly lowered metal–sulfur bond energy.^9^,^10^,^19^ Understanding how functional group selectivity emerges in 2-D TMDs catalysts is important, but insights remain limited. Here, we show that colloidal WS_2_ nanostructures containing single- and few-layer nanosheets, and without transition metal promotors, catalyze the selective hydrogenation of substituted nitroarenes to their corresponding aniline derivatives. Complementary microscopic and computational studies provide important insights into the origin of the catalytic selectivity on the TMD nanostructures, pointing to the important roles of atomic sulfur vacancies on the basal planes and tungsten-terminated edges. These WS_2_ nanostructures provide a well-defined platform to study how catalytic activity and selectivity can be achieved in nanostructured 2-D TMD materials.^20^

## Results and discussion

**Fig. 1:**
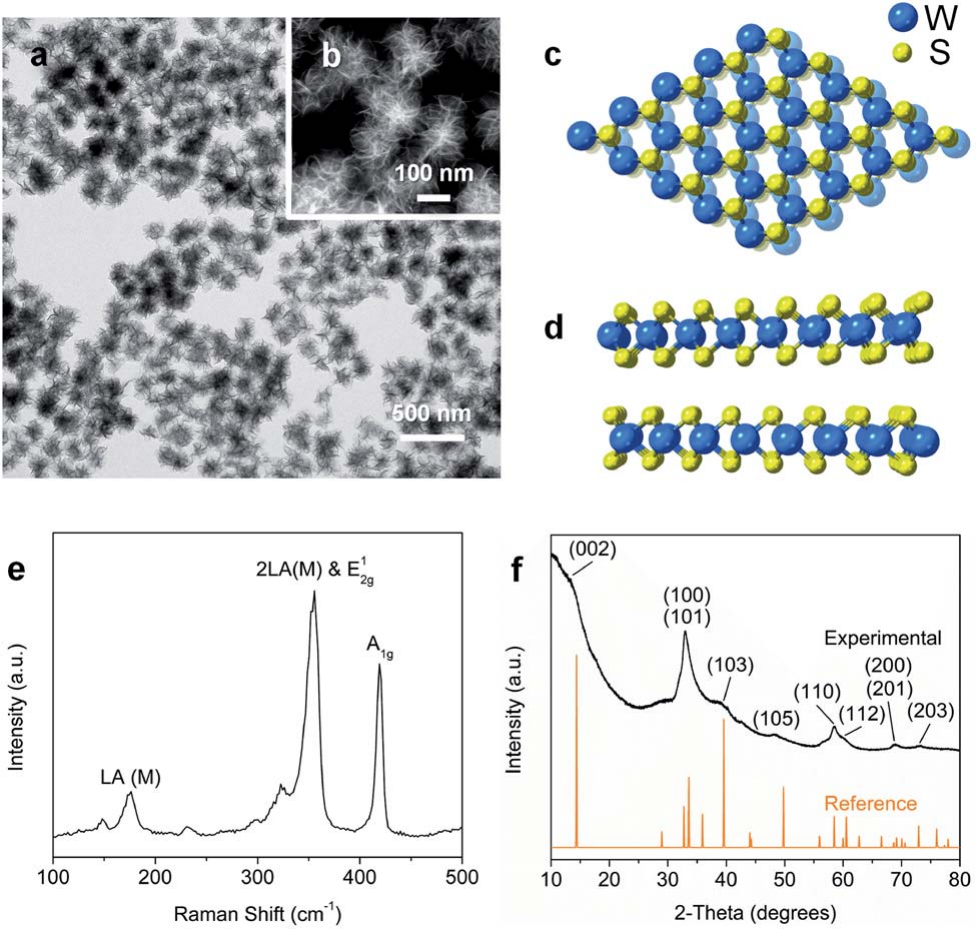
(a) TEM and (b) HAADF-STEM images of 2H-WS_2_ nanoflowers. (c) Top (basal plane) and (d) side (edge) views of the hexagonal structure for 2H-WS_2_. (e) Raman spectrum and (f) powder XRD data for the as-prepared 2H-WS_2_ nanostructures.

Nanostructured WS_2_ was synthesized in solution by reacting WCl_6_, hexamethyldisilazane, and CS_2_ in oleylamine and oleic acid.^21^ TEM (Fig. 1a) and HAADF-STEM (Fig. 1b) images of the resulting ∼100 nm colloidal nanostructures show that they contain nanosheets in a flower-like morphology.^22^ The crystal structure of 2H-WS_2_, shown in Fig. 1c and d, consists of alternating stacked monolayers that contain tungsten atoms coordinated by sulfur atoms in a trigonal prismatic geometry. The Raman spectrum in Fig. 1e shows the in-plane (E12g) and out-of-plane (A_1g_) vibration modes, as well as higher-order modes [LA(M) and 2LA(M)],^23^,^24^ which are characteristic of 2H-WS_2_ nanostructures and indicate the presence of defects.^25^ The XRD pattern in Fig. 1f shows further evidence for the formation of crystalline 2H-WS_2_. Polycrystallinity and strain arising from the curled nanosheets and small domain sizes result in a broad, yet characteristic and identifiable diffraction pattern that is consistent with 2H-WS_2_.^26^,^27^ STEM-EDS data reveal that the 2H-WS_2_ nanostructures are chalcogen deficient (Fig. S1†).^28^ XPS, which is sensitive to the chemical environment around each atom and can distinguish between the two WS_2_ polymorphs, confirms that the majority (∼90%) of the as-synthesized sample contains 2H-WS_2_, with the remainder being 1T-WS_2_ (Fig. S2 and Table S1†). The surface area of the flower-like 2H-WS_2_ nanostructures is estimated by BET to be 62.7 m^2^ g^–1^, which is much higher than that of the bulk powder sample (7.6 m^2^ g^–1^, Fig. S3†).

In a typical catalytic experiment, the 2H-WS_2_ nanostructures were added to a solution mixture containing THF, deionized water, and a substituted nitroarene. Hydrogenation was carried out for 8 h at 120 °C with 50 bar of H_2_, which are conditions comparable to previous studies of transition metal-based catalysts for analogous reactions.^15^,^16^,^29^ The model substrate was 3-nitrostyrene, which contains reducible nitro and vinyl groups. Using the aforementioned conditions, the 2H-WS_2_ nanoflowers facilitate >99% conversion of the nitro group to an amine and 94% retention of the vinyl group. In contrast, commercially available Pt/C unselectively hydrogenates both nitro and vinyl groups under the same conditions, while no detectable products were obtained with bulk WS_2_ powders (Table S2†). At lower H_2_ pressures (20 bar), 3-nitrostyrene can also be selectively transformed to 3-vinylaniline, though a longer reaction time (12 h) is required to achieve similar conversion (Fig. S4†).

**Fig. 2:**
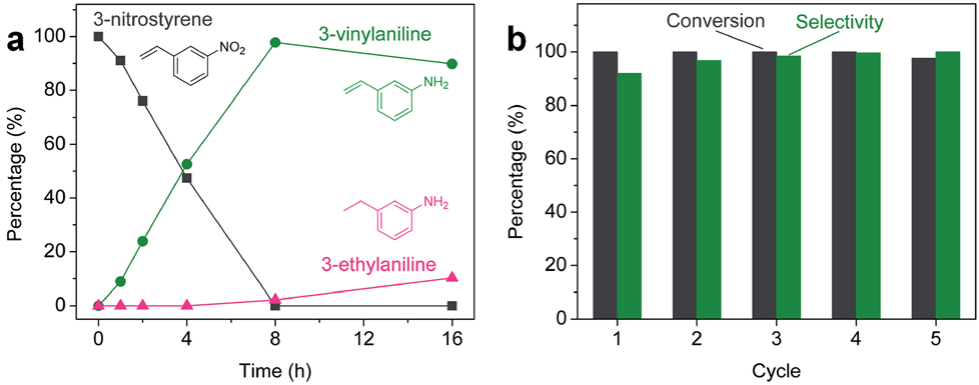
(a) Aliquot study for the selective hydrogenation of 3-nitrostyrene catalyzed by 2H-WS_2_ nanostructures at 50 bar H_2_ and 120 °C, showing the percentage of 3-nitrostyrene (grey), 3-vinylaniline (green), and 3-ethylaniline (pink) at different reaction times. (b) Percent conversion (grey) and selectivity (green) for the hydrogenation of 3-nitrostyrene to 3-vinylaniline using the same nanostructured 2H-WS_2_ catalyst over five successive cycles.

Aliquots taken during the reaction indicate that the conversion of 3-nitrostyrene to 3-vinylaniline at 120 °C with 50 bar of H_2_ is nearly linear with time up to 8 h, reaching >99% conversion and 98% selectivity (Fig. 2a). After 8 h, reduction of the vinyl group results in increasing formation of the 3-ethylaniline byproduct, and consequently a decrease in selectivity. Therefore, reduction of the nitro group takes place prior to that of the vinyl group, and adjusting experimental parameters can optimize both activity and selectivity. As shown in Fig. 2b, the 2H-WS_2_ catalysts remain highly active and selective over five consecutive hydrogenation reactions of 3-nitrostyrene, achieving 98% conversion with >99% selectivity on the fifth cycle and indicating a high degree of recyclability. TEM, XRD, EDS, and Raman data for 2H-WS_2_ nanostructures before catalysis and after one and five catalytic cycles are indistinguishable (Fig. S5 and S6†). XPS shows no change in the 2H : 1T phase ratio after 5 cycles. However, the W 4f and S 2p peaks exhibit a small shift (∼0.5 eV) towards lower binding energies after five cycles, suggesting that the surface of the nanostructured 2H-WS_2_ catalyst becomes slightly reduced after long-term reaction with hydrogen (Fig. S7†).

A broad scope of nitroarenes with diverse reducible groups including aldehydes, carboxylic acids, esters, amides, sulfonamides, nitriles, thioethers, ketones, halogens, and pyridines, as well as combinations of groups, were also tested for conversion to their corresponding anilines. As shown in Table 1, most substrates achieved complete conversion and >99% selectivity towards hydrogenation of the nitro group, leaving the other functional groups unaltered. Though most halide-containing anilines were obtained with >99% selectivity through hydrogenation of the corresponding nitro compounds under standard reaction conditions, a lower reaction temperature (100 °C) was used for 4-iodobenzene to avoid dehalogenation. Hydrogenation of 4-nitrobenzaldehyde to 4-aminobenzaldehyde could only be achieved with 78% selectivity, as hydrogenation of the aldehyde led to the formation of 4-methylaniline. In addition, nitrobenzene, nitrosobenzene, and *N*-phenylhydroxylamine were exclusively converted to aniline under standard reaction conditions, supporting the Haber mechanism where the nitro groups are converted to amines with nitroso and hydroxylamine intermediates.^30^

**Table 1:** Conversion and selectivity of nanostructured 2H-WS_2_ catalyzed hydrogenation of substituted nitroarenes to corresponding anilines

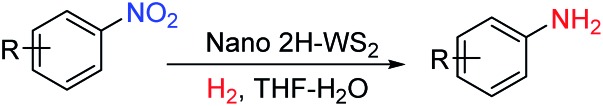			
Entry^*a*^	Substrate	Conv. (%)	Selec. (%)
1	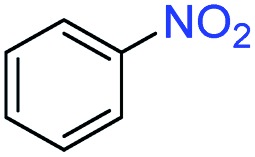	>99	>99
2	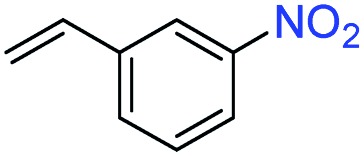	>99	94
3	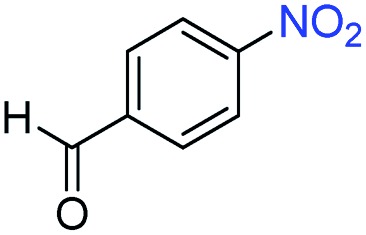	>99	78
4	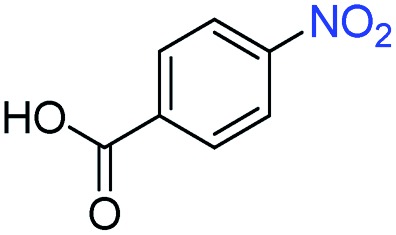	>99	>99
5	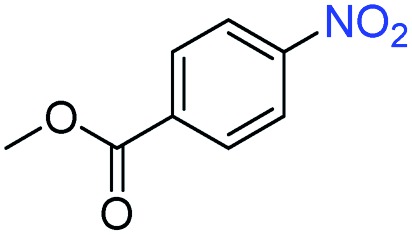	>99	>99
6	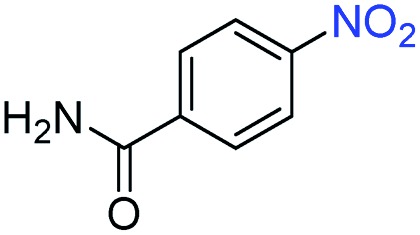	>99	>99
7	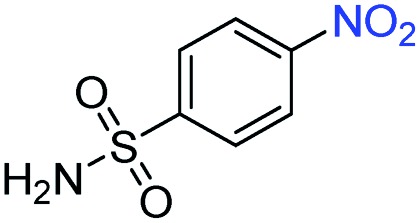	>99	>99
8	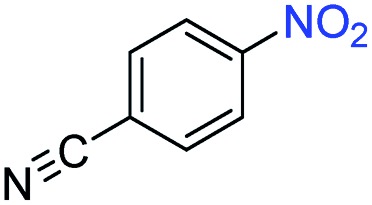	>99	>99
9	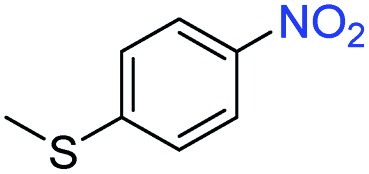	>99	>99
10	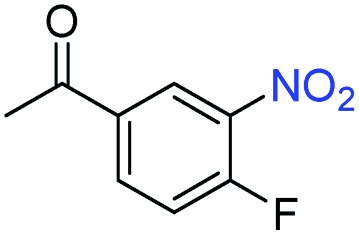	>99	>99
11	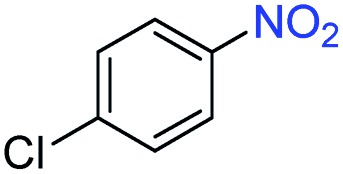	>99	>99
12	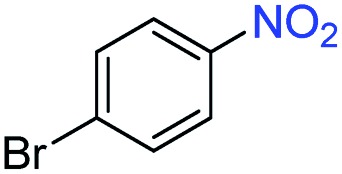	>99	>99
13^*b*^	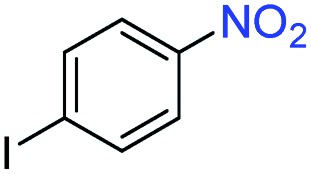	>99	>99
14	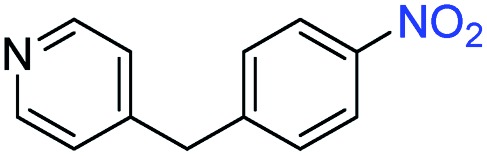	>99	>99
15	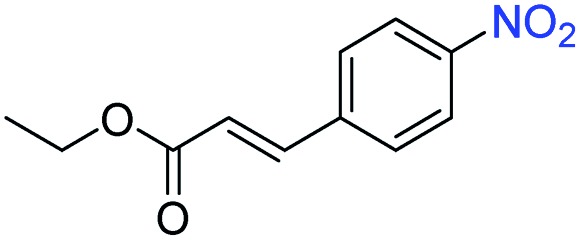	>99	>99

^*a*^Unless otherwise noted, hydrogenations were performed under the following conditions: WS_2_ (5 mg), THF (1 mL), H_2_O (125 μL), substrate (0.125 mmol), 8 h, 120 °C.

^*b*^Run at 100 °C.

**Fig. 3:**
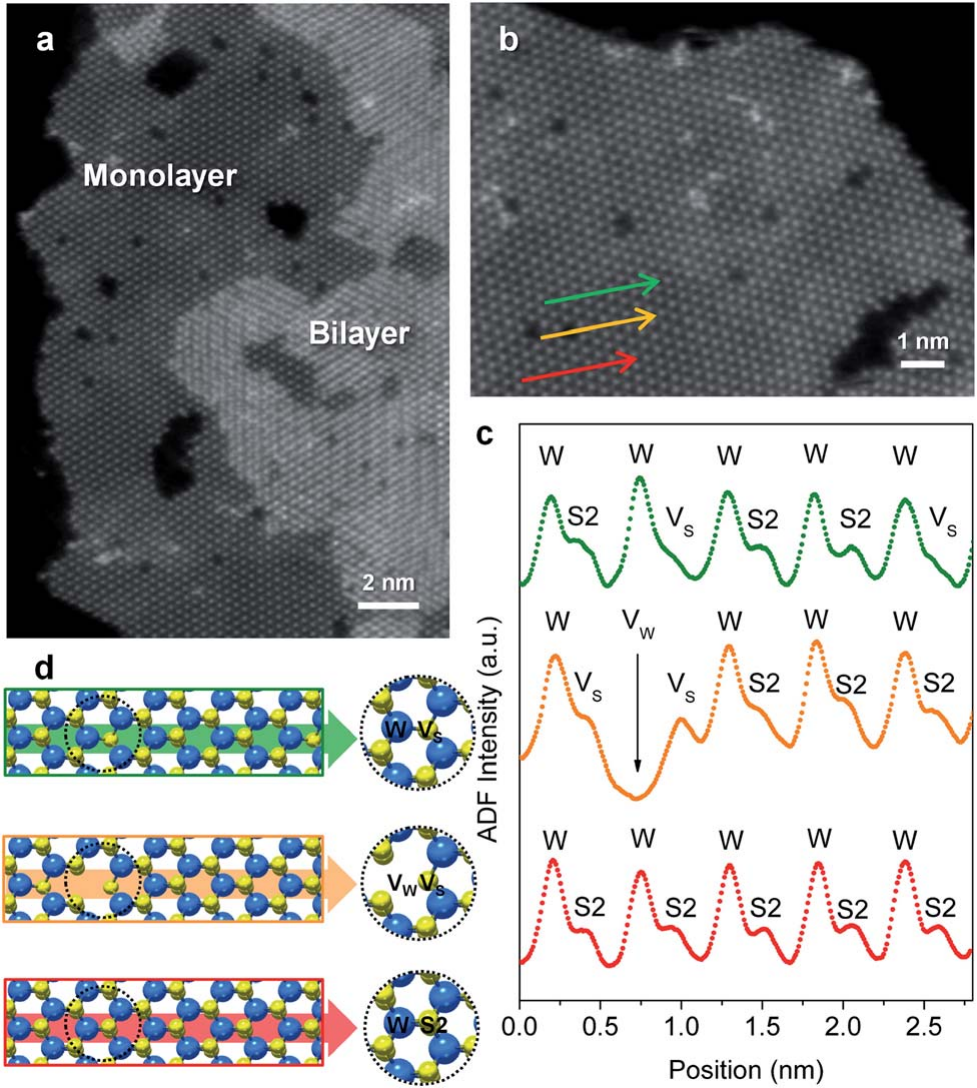
(a) High-resolution ADF-STEM image showing bilayer and monolayer domains with atomic vacancies on the surface (basal plane) of the 2H-WS_2_ nanostructures. (b) Atomically resolved ADF-STEM image for a region of monolayer 1H-WS_2_. (c) Experimental ADF intensity curves and (d) structural models corresponding to the three line scans indicated by the green, orange, and red arrows in (b), showing the alternation of tungsten (W) and sulfur (S_2_) configurations, as well as existence of tungsten (V_W_) and monosulfur (V_S_) vacancies.

Flower-like nanostructured WS_2_ is an active catalyst for selective hydrogenation while bulk WS_2_ shows no hydrogenation activity under analogous conditions (Table S2†). To understand how nanostructuring of this model 2-D TMD system enables catalysis, ADF-STEM was used to gain atomic-level insights into the structures of the as-prepared nanosheet catalysts. As shown in Fig. 3a, monolayer and bi-layer WS_2_ nanosheets were observed near the edges of the 2H-WS_2_ nanoflowers, with visible holes on the nanosheets indicating the presence of atomic vacancies. ADF-STEM can differentiate atoms based on *Z*-contrast (*Z* = atomic number), thus a line scan at high magnification can be analyzed to determine which atoms are in a particular location.^31^Fig. 3b–d show three line scans across the basal plane of a WS_2_ monolayer, highlighting a region with no vacancies, a region with an individual monosulfur vacancy (V_S_), and a region with a tungsten vacancy (V_W_) associated with a monosulfur vacancy (V_S_). While both tungsten and sulfur vacancies are present, STEM-EDS quantification reveals that the 2H-WS_2_ nanostructures are chalcogen-deficient, suggesting that sulfur vacancies dominate. Additional microscopic data also suggest that both tungsten- and sulfur-terminated edges are abundantly present in the flower-like nanostructures (Fig. S8†). Although not observed, disulfur vacancies (V_S2_) likely also exist, considering the sulfur vacancy concentration and previous reports.^23^

**Fig. 4:**
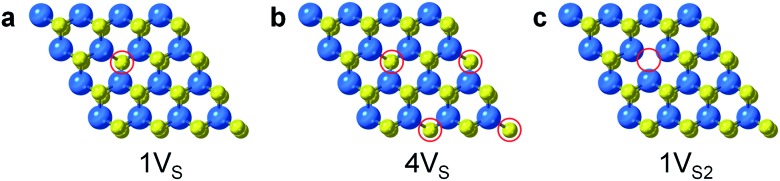
Simulated structures for different types of S-vacancies on the basal planes of 1H-WS_2_: (a) 1V_S_, (b) 4V_S_, and (c) 1V_S2_. The vacancy sites are highlighted by red circles.

Surface engineering of nanostructured TMD catalysts has been demonstrated as an effective approach to generate active edge and vacancy sites for hydrogen activation and production.^32^–^34^ Similar insights are important for selective hydrogenation reactions on TMDs in order to understand how selectivity emerges and therefore how it can be optimized, manipulated, and controlled. DFT calculations were performed to identify the optimized adsorption geometry of 3-nitrostyrene and the hydrogen transfer process on 2-D WS_2_ with different sulfur vacancy configurations. Monolayers of 1H-WS_2_ with a monosulfur vacancy (1V_S_), a disulfur vacancy (1V_S2_), and four evenly-distributed monosulfur vacancies (4V_S_) on a basal plane were constructed in a 4 × 4 × 1 supercell, corresponding to 3%, 6% and 12% sulfur vacancy concentrations, respectively (Fig. 4).

**Fig. 5:**
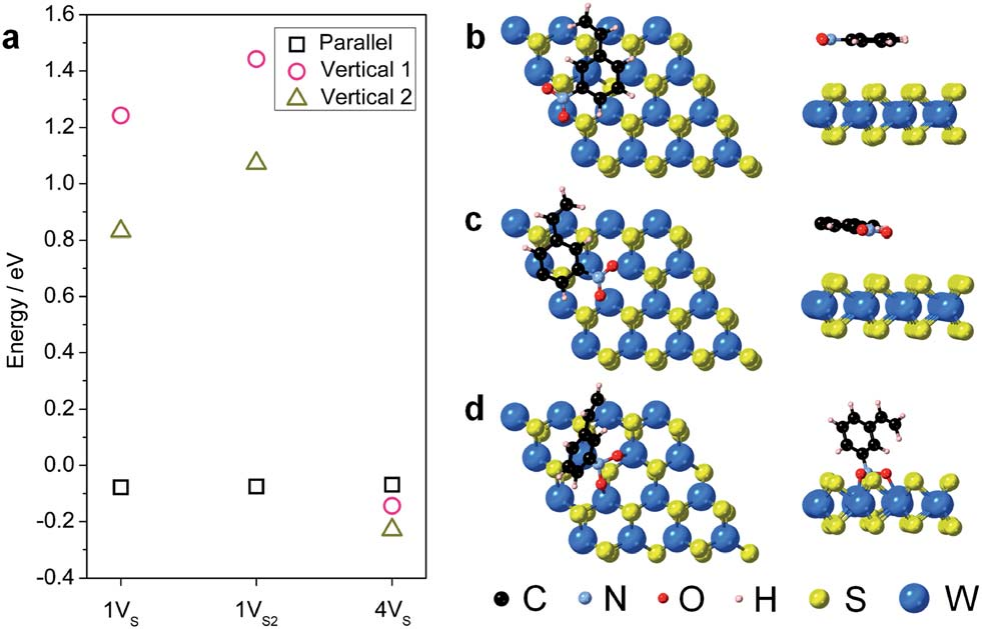
(a) Calculated adsorption energies of the three adsorption geometries (vertical 1, vertical 2, and parallel) on the three sulfur vacancy models (1V_S_, 1V_S2_, and 4V_S_) for 1H-WS_2_ basal planes. Lowest energy adsorbed structures of 3-nitrostyrene absorbed on the surface of a 1H-WS_2_ monolayer are shown for the (b) 1V_S_ (parallel); (c) 1V_S2_ (parallel); and (d) 4V_S_ (vertical) orientations on the different vacancy models.

During hydrogenation on a transition metal disulfide (MS_2_) catalyst, molecular hydrogen is adsorbed on the surface and is activated by either homolytic dissociation on the sulfur atoms to form two –S–H bonds or heterolytic dissociation to yield –S–H and –M–H species.^35^ The activated hydrogen atoms are then transferred to the adsorbed nitroarene molecule through active sites (*i.e.* vacancies and edge sites of nanostructured WS_2_), where the adsorption geometry of the nitroarene molecule plays a key role in determining the activity and selectivity. We thus first investigated the adsorption of 3-nitrostyrene on the 1V_S_, 1V_S2_, and 4V_S_ surfaces of WS_2_. Three adsorption geometries, including two vertical modes where the molecule stands up with the nitro group bound to the WS_2_ surface and one parallel mode where the molecule lies flat, parallel to the WS_2_ plane, were investigated (Fig. S9†).^36^,^37^ Calculation results suggest that the weakly adsorbed parallel configuration is preferable in the 1V_S_ and 1V_S2_ models, while the quasi-vertical configuration with two oxygen atoms from the nitro group bound to W atoms in the sulfur vacancy site is slightly preferred in the case of 4V_S_ (Fig. 5 and Table S3†). The positive adsorption energies in the 1V_S_ and 1V_S2_ models indicate that the vertical adsorbed states are unstable local minima. A high concentration of sulfur vacancies significantly promotes the vertical adsorption of 3-nitrostyrene on WS_2_. Favorable adsorption with a high concentration of vacancies could also lead to higher adsorbate coverages that motivate a vertical orientation due to surface crowding. This vertical orientation facilitates selective hydrogenation of the nitro group since it is positioned directly on the surface of the WS_2_ catalyst while the vinyl group does not interact with the surface.

For the 1V_S_ and 1V_S2_ models where 3-nitrostyrene preferentially adsorbs parallel to the WS_2_ surface and both the nitro and vinyl groups are oriented so that they could undergo catalytic hydrogenation, we carried out additional kinetic calculations involving stepwise hydrogenation of both the nitro and vinyl groups. We calculated the activation barriers (*E*_a_) and reaction energies (Δ*E*) for the hydrogenation of the nitro and vinyl groups, using the 1V_S_ model as a representative parallel-adsorption system. As summarized in Fig. S10 and S11,† the rate-determining step for hydrogenation of the nitro group is the transformation from R–NO_2_ to R–NOOH, which has a reaction barrier of 0.97 eV that is noticeably lower than that of the first step of the vinyl hydrogenation pathway (1.07 eV). Thus, selective hydrogenation of the nitro group is kinetically favored when there is a low concentration of sulfur vacancies.

**Fig. 6:**
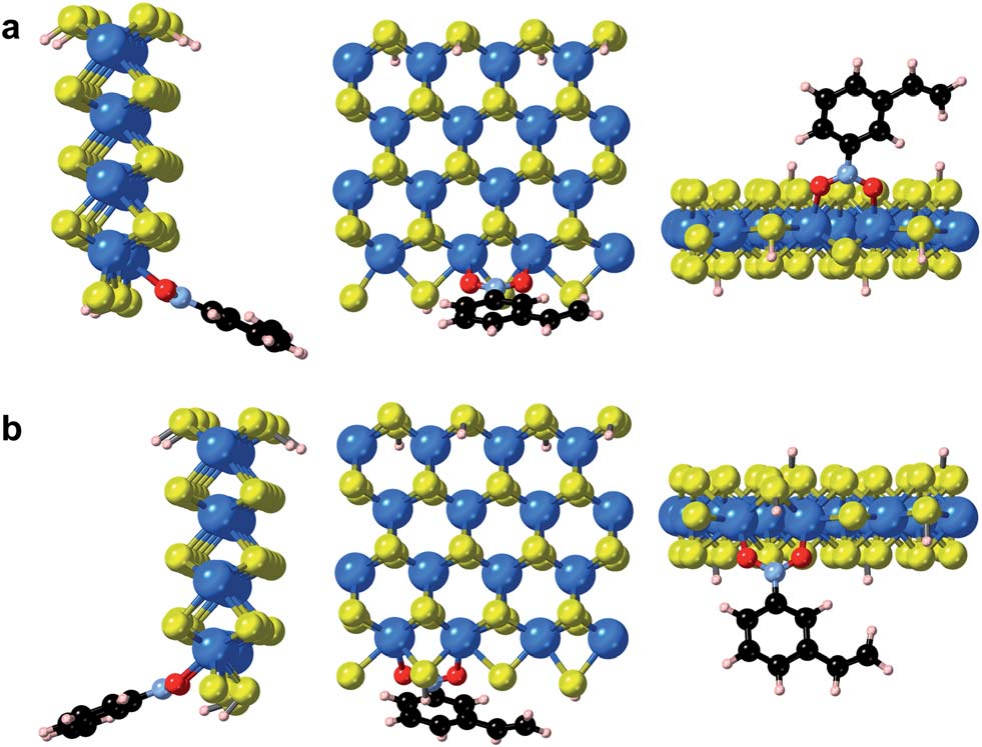
Optimized geometries of 3-nitrostyrene absorbed on the tungsten-terminated edges of 1H-WS_2_ monolayer are shown for the (a) W-2 and (b) W-3 models from three viewing angles.

In addition to point defects involving sulfur vacancies on the basal planes, the WS_2_ nanoflowers also provide abundant edge sites that could also serve as active sites for selective hydrogenation of 3-nitrostyrene. We therefore also calculated the adsorption energy of 3-nitrostyrene on the tungsten- and sulfur-terminated edges, which were constructed using 4 × 1 × 1 nanoribbons. Previous DFT calculations have demonstrated that, at high temperatures and partial pressures of H_2_, partial edge site coverage of sulfur and hydrogen adatoms is likely based on the previous *ab initio* thermodynamics methodology.^38^ Given the high pressures and temperatures utilized in our catalytic experiments, modeling of the tungsten-terminated edge sites included a coverage of *θ*_S_ = 0.5 and *θ*_H_ = 0.5, and the sulfur-terminated edge sites were modeled with *θ*_S_ = 1 and *θ*_H_ = 1 (Fig. S12†). As shown in Fig. 6, S13 and S14,† the nitro adsorption energies were calculated for five distinct configurations. Three adsorption geometries for the tungsten-terminated edge sites were considered, including head-on adsorption (W-1), the nitro group adsorbed to the tungsten atoms modified with one sulfur atom (W-2), and the nitro group adsorbed to the tungsten atoms modified with one sulfur atom and one hydrogen atom (W-3). Two geometries for the sulfur-terminated sites were also considered: head-on adsorption (S-1) and staggered head-on adsorption (S-2). Based on the optimized absorption energies, only the W-2 and W-3 configurations, involving the tungsten-terminated edge sites, revealed favorable adsorption of 3-nitrostyrene *via* a quasi-vertical geometry, where the two oxygen atoms from the nitro group are separately bonded to the two exposed tungsten atoms of WS_2_ (Fig. 6). For the other three geometries, either weak or unfavorable interactions were observed (Table S4†). For both the W-2 and W-3 orientations, the nitro group directly bonds to the tungsten atoms at the edges and results in selective hydrogenation, whereas the vinyl group does not directly interact with the WS_2_ nanostructures.

The mechanisms that result in selective hydrogenation of 3-nitrostyrene to 3-vinylaniline are different for regions of the nanostructured surface that have high *vs.* low sulfur vacancy concentrations and different edge terminations. However, the end result is the same – the nitro group is selectively hydrogenated relative to the vinyl group, and the selectivity arises from interactions between the substrate molecule and sulfur defects on the WS_2_ surface.

## Conclusions

Colloidally synthesized 2H-WS_2_ nanostructures, which have high surface areas and contain single- and few-layer nanosheets with high vacancy concentrations, serve as a model 2-D TMD system for understanding the origin of selectivity during hydrogenation reactions. The 2H-WS_2_ nanostructures catalyze the selective hydrogenation of substituted nitroarenes with molecular hydrogen, transforming them to the corresponding anilines in the presence of a broad scope of reducible functionalities. Microscopic and computational studies indicate that sulfur vacancies on the basal planes and tungsten-terminated edges facilitate chemoselectivity, where hydrogen preferentially transfers to the nitro group due either to the preferential vertical adsorption geometry at high basal-plane vacancy concentrations and tungsten-terminated edge sites, or to smaller kinetic barriers at lower basal-plane vacancy concentrations where parallel adsorption is favored. Such insights are important for guiding nanostructuring efforts in 2-D TMD catalyst systems, as well as other applications including responsive gas sensors, where vacancy-rich surfaces are common and defect engineering could tune adsorption behavior.^39^ Future efforts to synthesize more complex and precisely-tailored TMD nanostructures, including composition-tunable alloys and noble metal-anchored heterostructures,^28^,^40^–^42^ will provide a broader scope of systems to enable more comprehensive studies on the nature of the active sites, the various mechanism by which selectivity can be achieved and modified, and the role of interactions between active species and underlying substrates.

## Experimental

### Materials

Oleylamine (technical grade, 70%), tungsten(vi) chloride (WCl_6_, ≥99.9%, trace metal basis), hexamethyldisilazane (HMDS, reagent grade, ≥99%), carbon disulfide (CS_2_, ≥99.9%, anhydrous), tetrahydrofuran (THF, anhydrous, contains 250 ppm BHT as inhibitor, ≥99%), azobenzene (98%), 4-nitrobenzamide (98%), 4-nitrobenzenesulfonamide (97%), 4′-fluoro-3′-nitroacetophenone (97%), 3-nitrostyrene (96%), hydrazobenzene, 1-bromo-4-nitrobenzene (99%), ethyl 4-nitrocinnamate (predominately *trans*, 99%), 4′-aminoacetophenone (99%), 3-vinylaniline (contains KOH as inhibitor, 97%), 4-nitrothioanisole (96%), 1-chloro-4-nitrobenzene (99%), 1-iodo-4-nitrobenzene (98%), tetradecane (≥99%), nitrosobenzene (≥97%), 3-ethylaniline (98%), *p*-toluidine (99%), 4-(4-nitrobenzyl)pyridine (98%), 4-nitrobenzonitrile (97%), hydroxylamine hydrochloride (99%), azoxybenzene, and nitrobenzene (99%) were purchased from Sigma Aldrich. Oleic acid (technical grade, 90%) and 4-nitrobenzoic acid (99%) were purchased from Alfa Aesar. Methyl 4-nitrobenzoate (≥98.0%) and 4-nitrobenzaldehyde (≥98.0%) were purchased from TCI. 10% Pt on activated carbon powder was purchased from Premetek. Solvents, including hexane and isopropanol, were of analytical grade. All chemicals were used as received without further purification.

### Synthesis of 2H-WS_2_ nanostructures

The colloidal synthesis of the 2H-WS_2_ nanostructures was carried out according to the previously reported approach.^21^ 15 mL of oleylamine was added to a 100 mL three-neck flask and degassed for 30 min under vacuum at 120 °C. HMDS (0.5 mL) was then injected into the flask after cooling to 100 °C under argon and the mixture was heated to 320 °C. Meanwhile, WCl_6_ (50 mg, 0.125 mmol) was dissolved in 0.3 mL of oleic acid (0.95 mmol) and mixed with 5 mL of oleylamine (15.2 mmol) in an argon-flushed septum-capped vial. Upon injection, 0.24 mL of CS_2_ was introduced to the vial, forming a homogeneous solution that became hot due to the exothermicity of the reaction. The solution in the vial was subsequently injected dropwise into the three-neck flask at a rate of 10 mL h^–1^ using a syringe pump. After 30 min, the injection was stopped and the heating mantle was removed. After cooling down to room temperature, the products were washed three times with a 1 : 1 toluene/ethanol mixture, collected with centrifugation, and kept as a powder under argon.

### Hydrogenation reactions

In a typical reaction, 20 mg of the 2H-WS_2_ nanostructures in powder form was added to a septum-capped glass vial along with a Teflon-coated magnetic stir bar, 4 mL of THF, 0.5 mL of deionized water, and 0.5 mmol of substrate molecules. Sonication was carried out to make sure the dispersion was fully mixed. A 300 mL Parr stainless steel autoclave (4766-T-SS-3000-VGR) was used for all hydrogenation reactions. The septum cap of every sample was punctured with a disposable 18 gauge needle to allow the partitioning of H_2_ into the vial. Once prepared and sealed, the autoclave was flushed twice with H_2_ gas at 25 bar to remove ambient air, and then filled to the final pressure (50 bar H_2_). Once pressurized, the autoclave was heated to 120 °C and held at that temperature for 8 h. The reaction was cooled to room temperature, the H_2_ atmosphere was discharged, and the samples were prepared for GC-MS as described below. For the hydrogenation of substituted nitroarenes, a smaller scale with 5 mg of 2H-WS_2_ nanostructures in powder form, 1 mL of THF, 0.125 mL of deionized water, and 0.125 mmol of substrate molecules were utilized.

### GC-MS analysis

Typically, tetradecane (100 μL for 0.5 mmol scale reactions, 25 μL for 0.125 mmol scale reactions) was injected into the reaction mixture post-reaction as an internal standard. The catalyst from the reaction mixture was then removed *via* centrifugation at 13 500 rpm and the resulting solution was diluted with THF and submitted for GC-MS analysis. Product yields were determined *via* calibration curves made using commercially available reaction products. If no substrate signal was observed *via* GC-MS, it was assumed that the reaction had proceeded to >99% conversion. Similarly, if the desired aniline was observed as the only product, it was assumed that the reaction had proceeded with >99% selectivity.

### Recycling experiments

The hydrogenation of 0.5 mmol 3-nitrostyrene using 20 mg WS_2_ nanostructures as the catalyst was performed as described above. Following the completion of the reaction, 100 μL tetradecane was added to the reaction mixture. The WS_2_ nanostructures were separated from the reaction mixture *via* centrifugation, and the resulting supernatant was diluted with THF and submitted for GC-MS analysis to determine reaction yields, as described above. The remaining WS_2_ nanostructures were washed and centrifuged twice with THF, and then used as the catalyst for another hydrogenation of 3-nitrostyrene (0.5 mmol scale). This process was repeated for a total of five times to evaluate the catalyst recyclability.

### Characterization

Powder X-ray diffraction (XRD) patterns were collected using a Bruker-AXS D8 Advance diffractometer equipped with Cu Kα radiation and a LynxEye 1-D detector. Simulated XRD patterns of 2H-WS_2_ were generated using the CrystalMaker/CrystalDiffract software package. Transmission electron microscopy (TEM), high-angle annular dark-field scanning transmission electron microscopy (HAADF-STEM) images, and energy dispersive X-ray spectroscopy (EDS) data with element maps were acquired using a FEI Talos F200X operating at 200 kV. High-resolution ADF-STEM images were obtained using a FEI Titan^3^ G2 60/300 TEM with a spherical aberration corrector on both the probe- and the image-forming lens at an accelerating voltage of 80 kV. ADF line scan was obtained using ImageJ software. Bruker ESPRIT 2 software was applied for EDS data interpretation. Micro-Raman measurements were performed in a Renishaw inVia confocal microscope-based Raman spectrometer with 514.5 nm laser. The laser spot size using the 100× objective lens was approximately 1 μm. X-ray photoelectron spectroscopy (XPS) experiments were performed using a Physical Electronics VersaProbe II instrument equipped with a monochromatic Al Kα X-ray source (*hν* = 1486.7 eV) and a concentric hemispherical analyzer. Charge neutralization was performed using both low energy electrons (<5 eV) and argon ions. The binding energy axis was calibrated using sputter cleaned Cu foil (Cu 2p_3/2_ = 932.7 eV, Cu 3p_3/2_ = 75.1 eV). Peaks were charge referenced to the CH_*x*_ peak in the carbon 1s spectra at 284.8 eV. Measurements were made at a takeoff angle of 45° with respect to the sample surface plane. This resulted in a typical sampling depth of 3–6 nm (95% of the signal originated from this depth or shallower). Quantification was carried out using instrumental relative sensitivity factors (RSFs) that account for the X-ray cross section and inelastic mean free path of the electrons. Nitrogen adsorption analysis was carried at 77 K with an Accelerated Surface Area and Porosimetry Analyzer (ASAP 2020; Micromeritics Instrument Corp.). Prior to the measurement, the sample was degassed at 333 K for 24 h under 4 μm Hg vacuum. Surface area was estimated using the Brunauer, Emmett and Teller (BET) equation.^43^

### Calculations

Density functional theory (DFT) calculations were performed using Vienna *ab initio* simulation package (VASP) 5.4.4.^44^ The projector-augmented-wave (PAW) approach was utilized to treat the core electrons,^45^,^46^ and the Perdew–Burke–Ernzerhof (PBE) exchange–correlation functional of the generalized gradient approximation (GGA) was used to describe electron interactions.^47^ Kinetic cut-off energy for the plane-wave basis set was set as 450 eV. Gaussian smearing method was applied with smearing width of 0.01 eV.

Monolayer WS_2_ with trigonal prismatic coordination (1H-WS_2_) was selected as the surface slab model for the calculation of molecule adsorption and reaction pathways. Considering the large steric hinderance of 3-nitrostyrene, we applied a 4 × 4 × 1 supercell to prevent the potential intermolecular repulsion effect induced by coverage. Three distinct types of sulfur vacancies were modeled: only one sulfur atom removed from the lattice (denoted as 1V_S_); both top and bottom sulfur atoms removed (denoted as 1V_S2_); and four sulfur vacancies uniformly distributed on one side of the monolayer structure (denoted as 4V_S_). The vacuum space between periodic images is at least 12 Å to minimize the interactions between adjacent images. For this supercell, a 1 × 1 × 1 Γ-centered *k*-point mesh was applied. We tested the convergence with 3 × 3 × 1 *k*-point mesh and found that for selected adsorption and reaction, the energy difference between the results obtained with two meshes was less than 0.05 eV. In the case of 3-nitrostyrene adsorbed on tungsten- and sulfur-terminated edges, calculations were performed with a 3 × 1 × 1 gamma-centered *k*-point due to the one-dimensional structure. The electronic convergence criteria was set as 1 × 10^–6^ eV. Structural optimization was considered complete when the magnitude of the forces on the atoms was less than 0.01 eV Å^–1^, while force convergence was set as 0.05 eV Å^–1^ for the search of the transition states. The transition states located using the climbing image nudged elastic band (CI-NEB) method were further refined using the dimer method with a force criteria of 0.01 eV Å^–1^.^48^,^49^ Transition states were confirmed by an imaginary frequency corresponding to the reactive mode. In some cases, there was another small imaginary frequency corresponding to the rocking vibration mode of the benzene group. However, due to the tight force convergence, further energy gains to eliminate the small imaginary frequency for the transition states would be low.

## Conflicts of interest

There are no conflicts to declare.

## Supplementary Material

Supplementary information
