# Supplementary material for: Defect-mediated selective hydrogenation of nitroarenes on nanostructured WS2
Source: Chem Sci. 2019 Sep 19;10(44):10310–7. doi: 10.1039/c9sc03337h (PMC6979393; doi:10.1039/c9sc03337h)
Supplement: Supplementary file 1 [file SC-010-C9SC03337H-s001.pdf]

Electronic Supplementary Material (ESI) for Chemical Science.
This journal is © The Royal Society of Chemistry 2019

Defect-mediated selective hydrogenation of nitroarenes on nanostructured WS_2

Yifan Sun,‡[a,e] Albert J. Darling,‡[a] Yawei Li,[b] Kazunori Fujisawa,[c,e] Cameron F. Holder,[a] He Liu,[a] Michael J. Janik,*[b] Mauricio Terrones,*[a,c,d,e] Raymond E. Schaak*[a,b]

[a] Department of Chemistry and Materials Research Institute, The Pennsylvania State University, University Park, PA 16802, USA. E-mail of R.E.S.: res20@psu.edu

[b] Department of Chemical Engineering, The Pennsylvania State University, University Park, PA 16802, USA. E-mail of M.J.J.: mjj13@psu.edu

[c] Department of Physics, The Pennsylvania State University, University Park, PA 16802, USA. E-mail of M.T.: mut11@psu.edu

[d] Department of Materials and Science Engineering, The Pennsylvania State University, University Park, PA 16802, USA

[e] Center for 2-Dimensional and Layered Materials, The Pennsylvania State University, University Park, PA 16802, USA

‡ These authors have contributed equally to this study.

Supplementary Tables

Table S1 Relative percentages of 1T- and 2H-WS_2 estimated through integration of the $W4f_{7/2}$ peak for the three samples, which indicates no noticeable phase transition is observed through the hydrogenation process. Without adding HMDS, we also prepared the 1T-WS_2 samples,[1] which yielded low conversion when tested for the hydrogenation of 3-nitrostyrene under the same conditions.

	1T Phase Percentage / %	2H Phase Percentage / %
**W region (fresh)**	8.5	91.5
**W region (after 5 cycles)**	12.2	87.8
**S region (fresh)**	14.8	85.2
**S region (after 5 cycles)**	11.3	88.7

Table S2 Hydrogenation performance of 3-nitroarenes for 8 h at 120 °C with 50 bar of H_2 using commercial Pt/C catalyst, bulk 2H-WS_2 powder, and 1T-WS_2 sample.

	Conversion / %	Selectivity / %
**Pt/C**	>99	<1
**Bulk 2H-WS_2**	<1	N/A
**Colloidal 1T-WS_2**	18	>99

Table S3 DFT-optimized adsorption energies of three adsorption geometries (vertical 1, vertical 2 and parallel) on the three sulfur vacancy models ($1V_S$, $1V_{S2}$ and $4V_S$).

	Vertical 1 / eV	Vertical 2 / eV	Parallel / eV
**$1V_S$**	1.243	0.831	-0.078
**$1V_{S2}$**	1.442	1.073	-0.075
**$4V_S$**	-0.144	-0.229	-0.069

Table S4 DFT-optimized adsorption energies of 3-nitrostyrene adsorbed on the tungsten- and sulfur-terminated edge models (W-1, W-2, W-3, S-1 and S-2).

	Adsorption Energy / eV
**W-1**	-0.047
**W-2**	-0.554
**W-3**	-0.694
**S-1**	1.630
**S-2**	0.004

Supplementary Figures

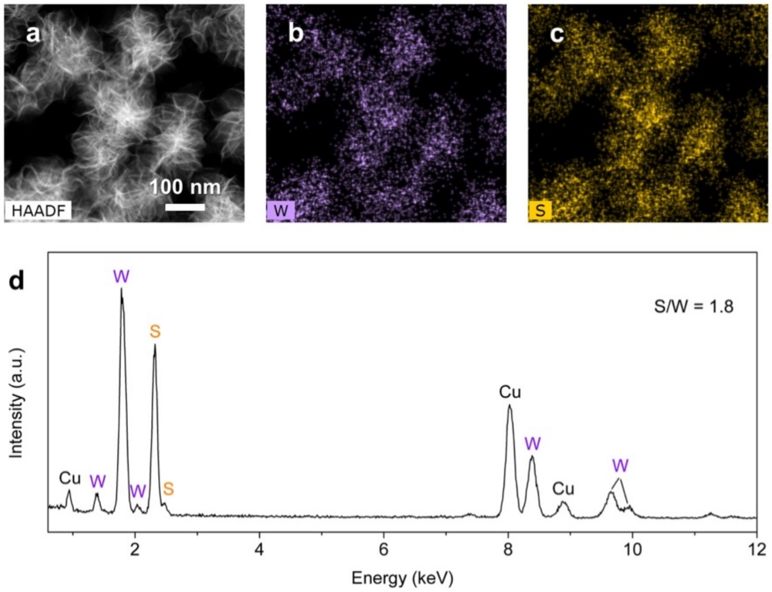

Fig. S1 (a) HAADF-STEM image, STEM-EDS element maps for (b) W and (c) S, and (d) EDS spectra for the as-synthesized 2H-WS_2 nanostructures. EDS quantification reveals a S:W ratio of 1.8, indicating that the material is sulfur deficient. The Cu signal comes from the Cu TEM grids.

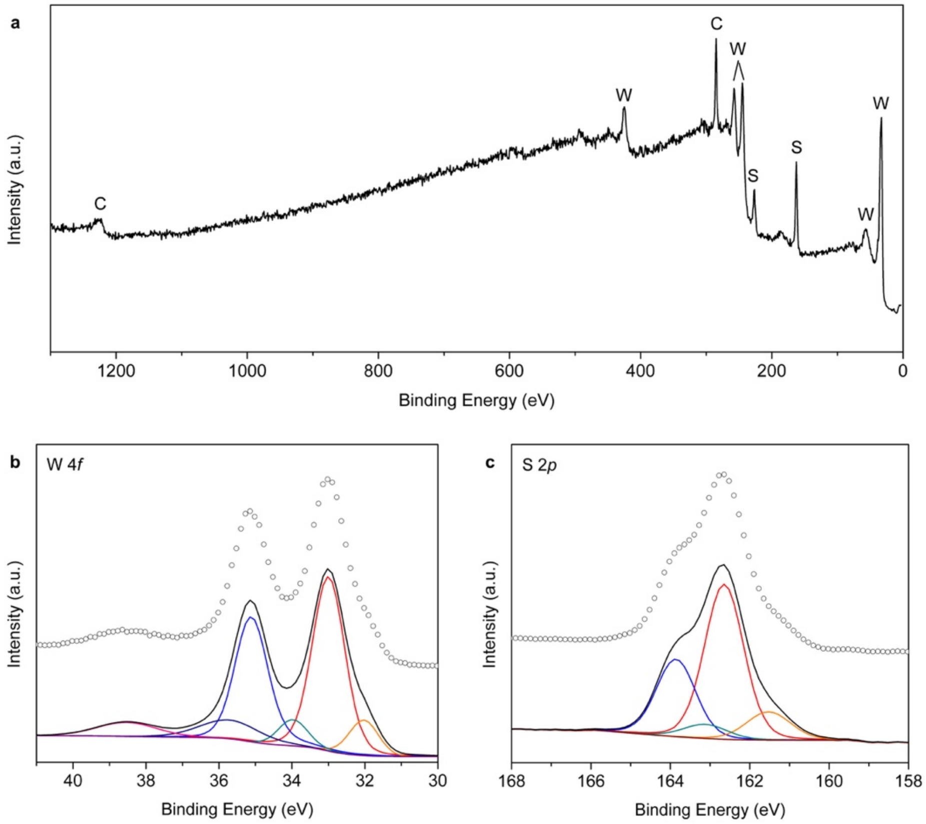

Fig. S2 (a) XPS survey scans, and high-resolution XPS spectra showing the (b) W 4*f* and (c) S 2*p* regions for the as-synthesized 2H-WS_2 nanostructures.

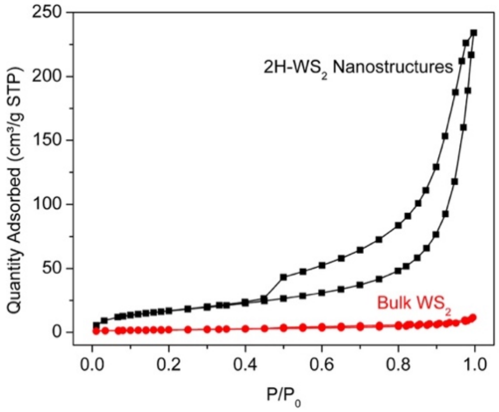

Fig. S3 N_2 adsorption isotherms for bulk and nanostructured 2H-WS_2. The surface area of the 2H-WS_2 nanostructures is estimated to be 62.7 m^2/g, higher than that for the bulk powder sample (7.6 m^2/g).

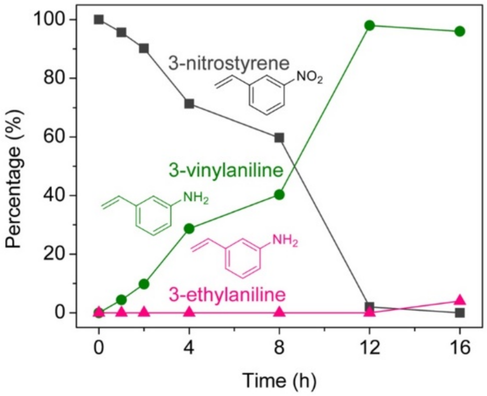

Fig. S4 Aliquot study for the selective hydrogenation of 3-nitrostyrene catalyzed by 2H-WS_2 nanostructures at 20 bar H_2 and 120 °C, indicating 3-nitrostyrene can also be selectively hydrogenated with 2H-WS_2 nanostructures at lower pressures, but with a longer reaction time.

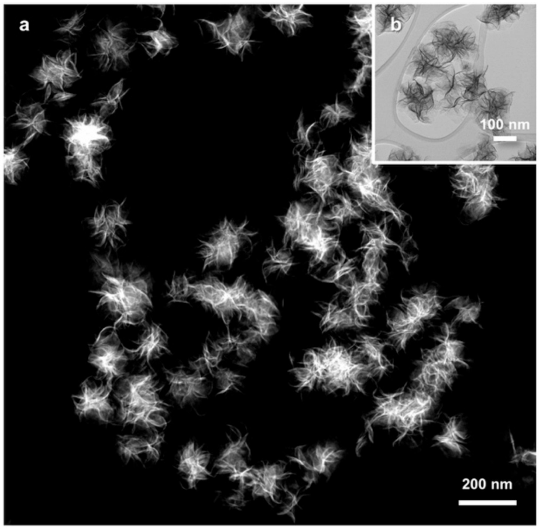

Fig. S5 (a) HAADF-STEM and (b) TEM images for the retrieved nanostructured 2H-WS_2 catalysts after one cycle.

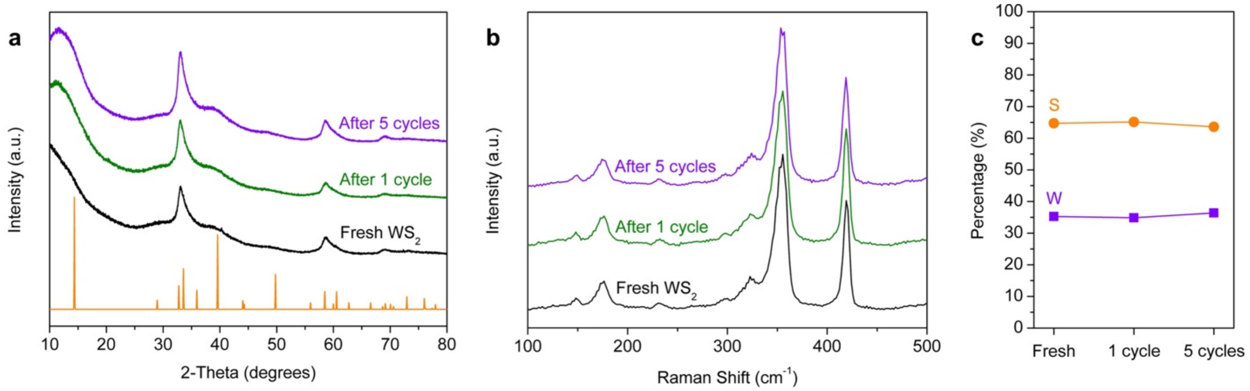

Fig. S6 (a) Powder XRD patterns, (b) Raman spectra, and (c) STEM-EDS quantifications for the as-synthesized 2H-WS_2 nanostructures, nanostructured 2H-WS_2 catalysts retrieved after one cycle, and nanostructured 2H-WS_2 catalysts retrieved after five successive cycles.

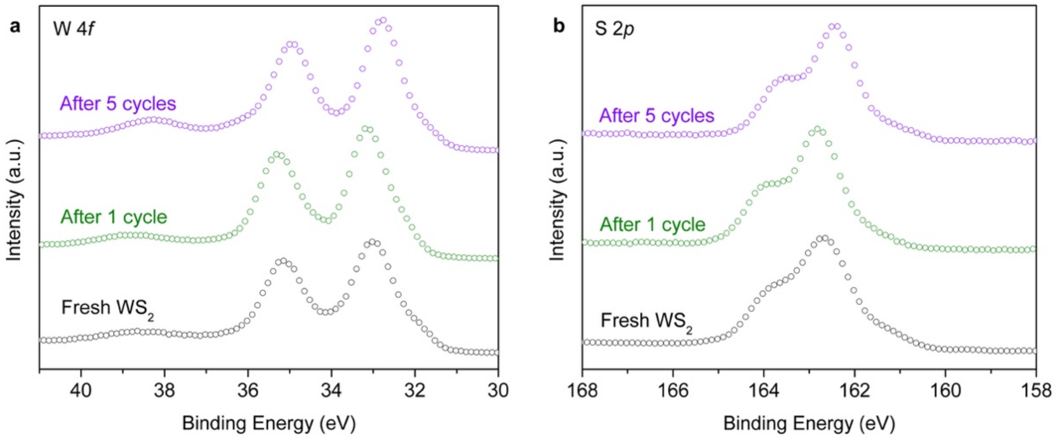

Fig. S7 High-resolution XPS spectra showing the (a) W 4*f* and (b) S 2*p* regions for the as-synthesized 2H-WS_2 nanostructures, nanostructured 2H-WS_2 catalysts retrieved after one cycle, and nanostructured 2H-WS_2 catalysts retrieved after five successive cycles. Peak shifts towards lower energies for both S and W suggest that the surface becomes slightly reduced during repeated hydrogenation processes.

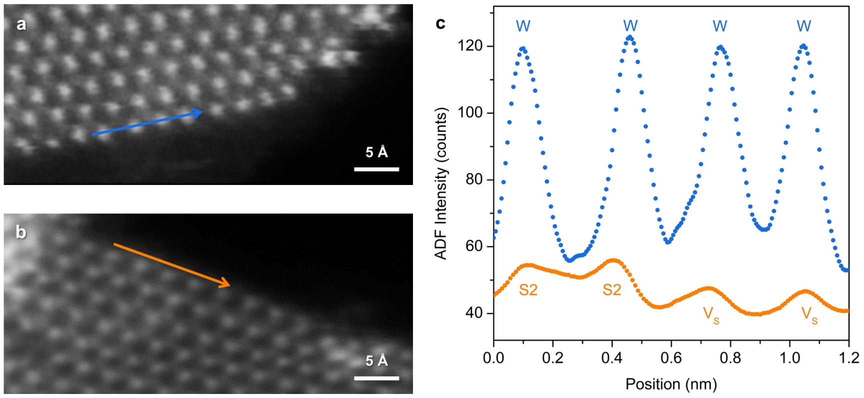

Fig. S8 Atomic-resolution ADF-STEM images of (a) tungsten- and (b) sulfur-terminated edges. (c) Experimental ADF intensity curves corresponding to the line scans indicated by the blue and orange arrow in (a) and (b), respectively, showing the existence of W at the tungsten-terminated edge, as well as S2 and V_S vacancies, in the sulfur-terminated edge.

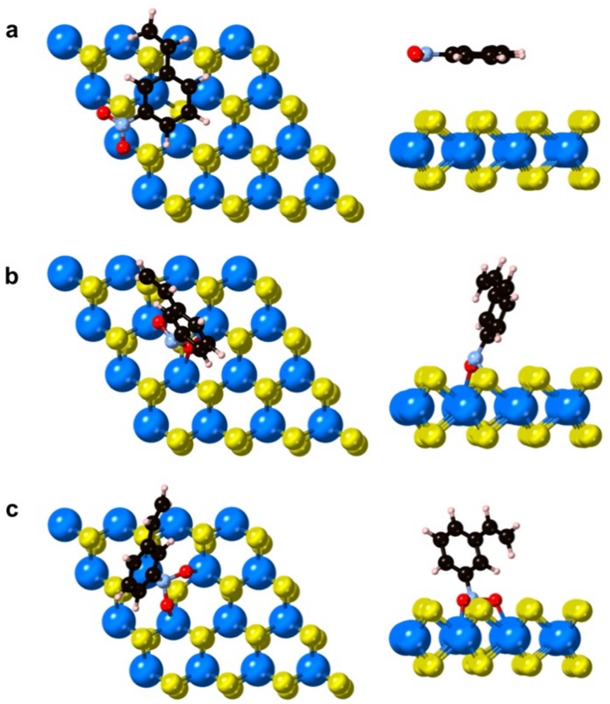

Fig. S9 The three basal plane adsorption geometries of 3-nitrostyrene on the $1V_S$ vacancy models: (a) parallel, (b) vertical 1, and (c) vertical 2.

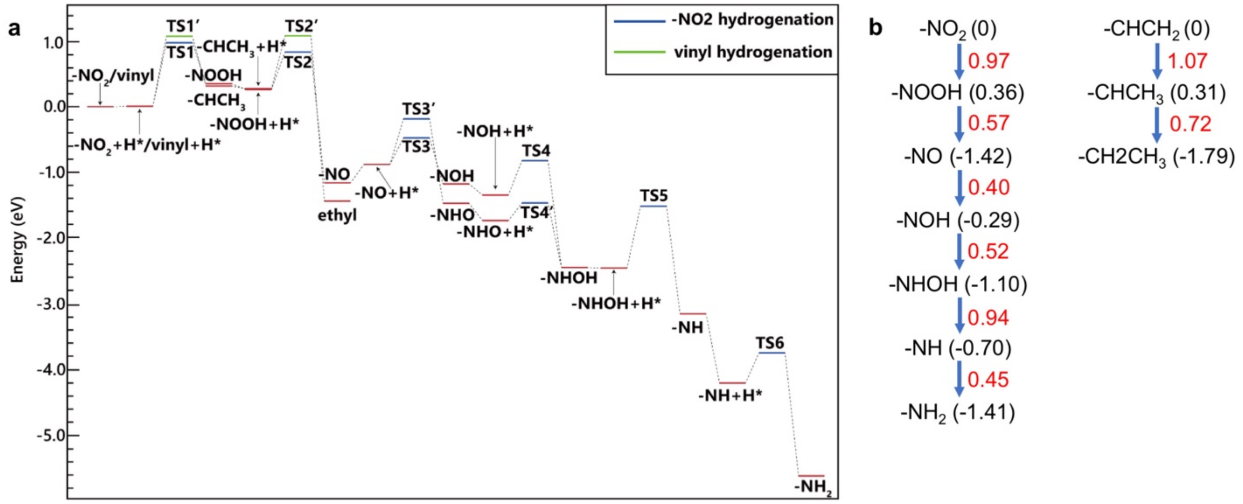

Fig. S10 (a) Full reaction routes for the hydrogenation of nitro ($-NO_2$) and vinyl ($-CH=CH_2$) groups of 3-nitrosyrene on the $1V_S$ vacancy model of $1H-WS_2$ basal planes. Initial and final states, as well as reaction intermediates, are denoted by solid red lines, whereas transition states are denoted by solid blue and green lines. (b) Calculated reaction energies (number in parenthesis in black color) and activation barriers (number in red color) for the hydrogenation of nitryl and vinyl groups on the $1V_S$ vacancy model of monolayer $1H-WS_2$.

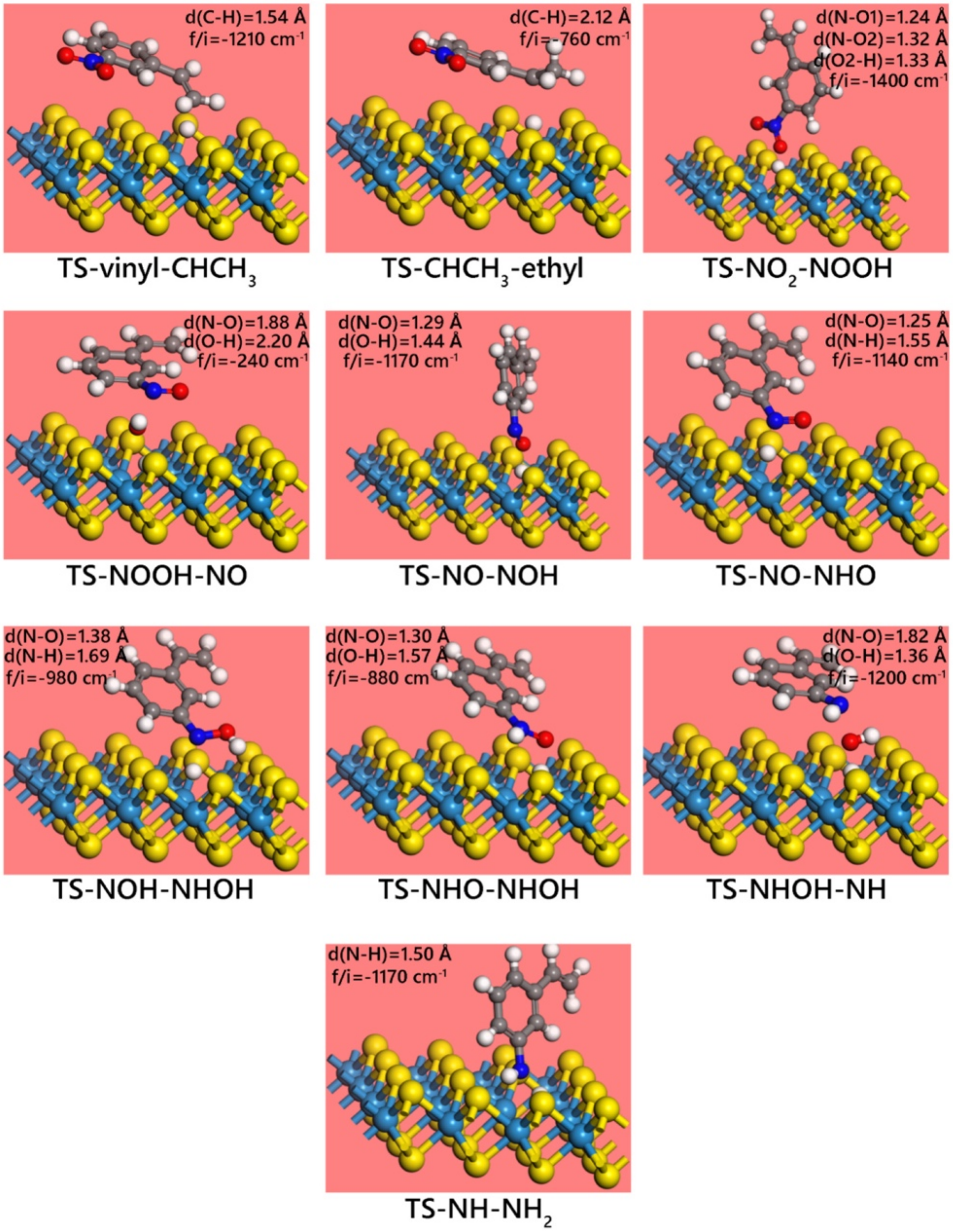

Fig. S11 Optimized structure with magnitude of the imaginary frequency for the transition states shown in Figs. S10a and S10b.

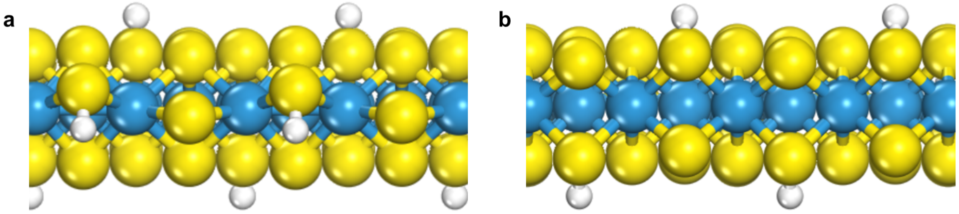

Fig. S12 (a) Simulated structure for tungsten-terminated edges modified with $\theta_S = 0.5$ and $\theta_H = 0.5$. (b) Simulated structure for sulfur-terminated edges modified with $\theta_S = 1$ and $\theta_H = 1$. Coverage of sulfur (θ_S) and hydrogen (θ_H) on the WS_2 edges are defined according to Rosen et al.[2]

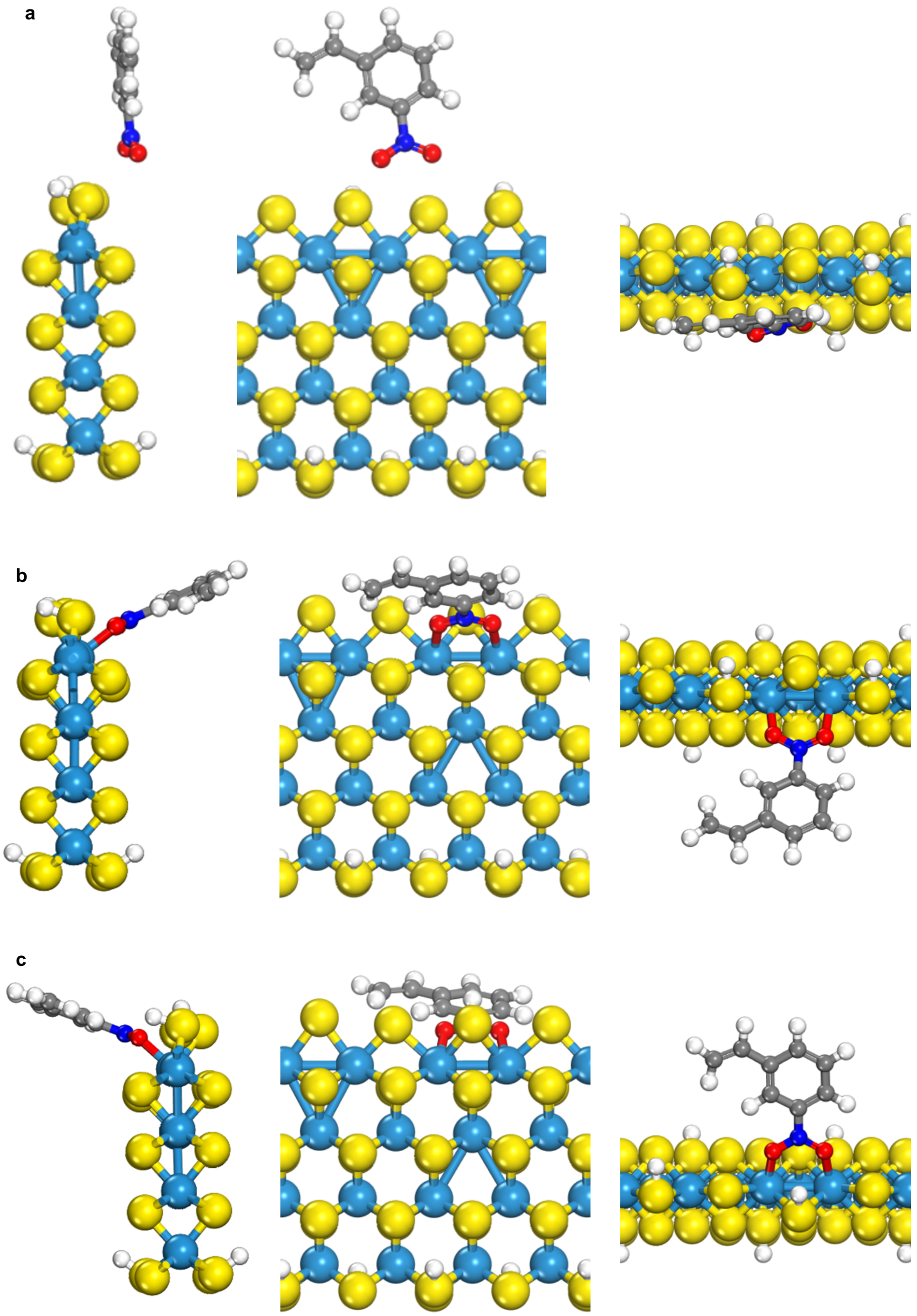

Fig. S13 Optimized geometries of 3-nitrostyrene absorbed on the tungsten-terminated edge models (a) W-1, (b) W-2, and (c) W-3 from three viewing angles.

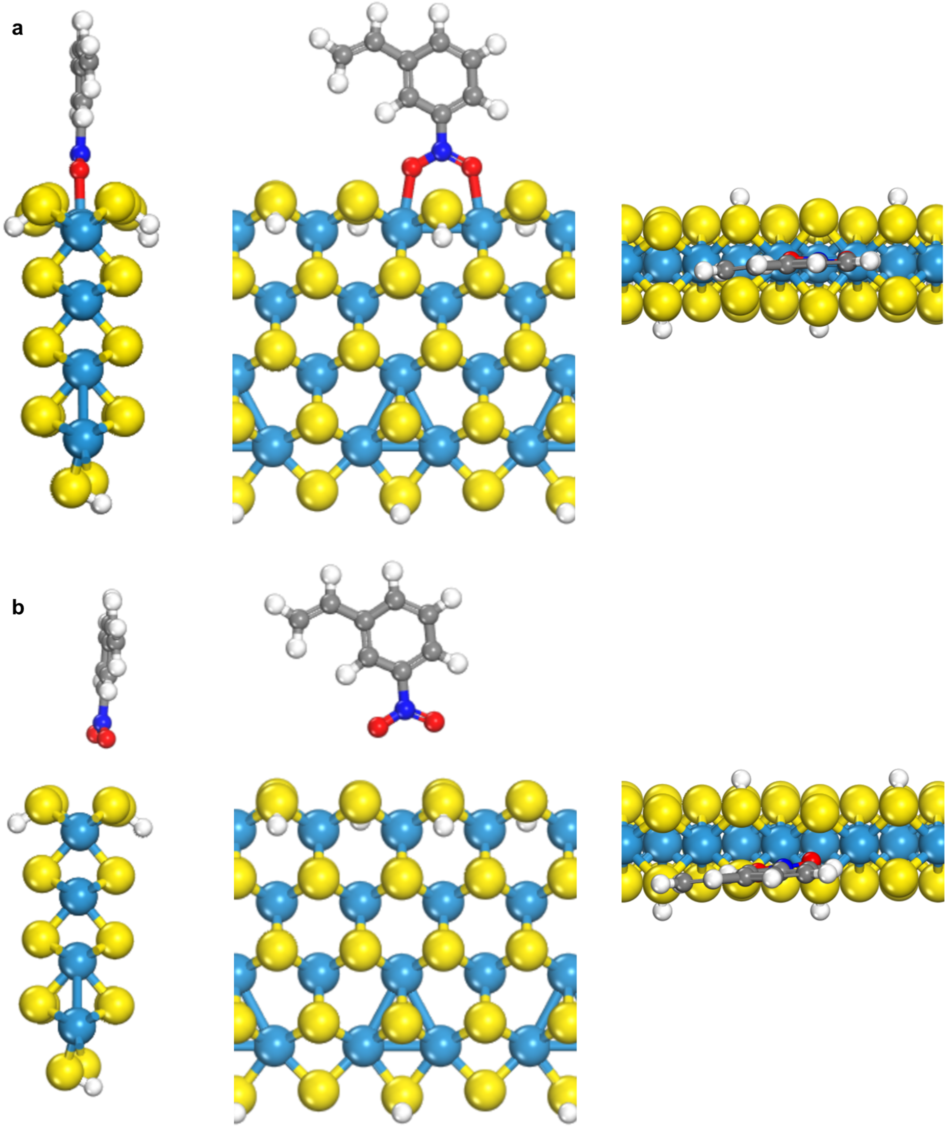

Fig. S14 Optimized geometries of 3-nitrostyrene adsorbed on the sulfur-terminated edge models (a) S-1 and (b) S-2 from three viewing angles.

References

1. B. Mahler, V. Hoepfner, K. Liao and G. A. Ozin, *J. Am. Chem. Soc.*, 2014, **136**, 14121–14127.

2. A. S. Rosen, J. M. Notestein and R. Q. Snurr, *J. Phys. Chem. C*, 2018, **122**, 15318–15329.
